# DEK deficiency suppresses mitophagy to protect against house dust mite-induced asthma

**DOI:** 10.3389/fimmu.2023.1289774

**Published:** 2024-01-11

**Authors:** Qiaoyun Bai, Ruobai Liu, Changlin Quan, Xue Han, Dandan Wang, Chongyang Wang, Zhiguang Wang, Li Li, Liangchang Li, Hongmei Piao, Yilan Song, Guanghai Yan

**Affiliations:** ^1^ Jilin Key Laboratory for Immune and Targeting Research on Common Allergic Diseases, Yanbian University, Yanji, China; ^2^ Department of Anatomy, Histology and Embryology, Yanbian University Medical College, Yanji, China; ^3^ Department of Respiratory Medicine, Affiliated Hospital of Yanbian University, Yanji, China

**Keywords:** asthma, DEK, PINK1-Parkin, mitophagy, NLRP3 inflammasome

## Abstract

DEK protein is highly expressed in asthma. However, the mechanism of DEK on mitophagy in asthma has not been fully understood. This study aims to investigate the role and mechanism of DEK in asthmatic airway inflammation and in regulating PINK1-Parkin-mediated mitophagy, NLRP3 inflammasome activation, and apoptosis. PINK1-Parkin mitophagy, NLRP3 inflammasome, and apoptosis were examined after gene silencing or treatment with specific inhibitors (MitoTEMPO, MCC950, and Ac-DEVD-CHO) in house dust mite (HDM) or recombinant DEK (rmDEK)-induced WT and DEK-/- asthmatic mice and BEAS-2B cells. The regulatory role of DEK on ATAD3A was detected using ChIP-sequence and co-immunoprecipitation. rmDEK promoted eosinophil recruitment, and co-localization of TOM20 and LC3B, MFN1 and mitochondria, LC3B and VDAC, and ROS generation, reduced protein level of MnSOD in HDM induced-asthmatic mice. Moreover, rmDEK also increased DRP1 expression, PINK1-Parkin-mediated mitophagy, NLRP3 inflammasome activation, and apoptosis. These effects were partially reversed in DEK^-/-^ mice. In BEAS-2B cells, siDEK diminished the Parkin, LC3B, and DRP1 translocation to mitochondria, mtROS, TOM20, and mtDNA. ChIP-sequence analysis showed that DEK was enriched on the ATAD3A promoter and could positively regulate ATAD3A expression. Additionally, ATAD3A was highly expressed in HDM-induced asthma models and interacted with DRP1, and siATAD3A could down-regulate DRP1 and mtDNA-mediated mitochondrial oxidative damage. Conclusively, DEK deficiency alleviates airway inflammation in asthma by down-regulating PINK1-Parkin mitophagy, NLRP3 inflammasome activation, and apoptosis. The mechanism may be through the DEK/ATAD3A/DRP1 signaling axis. Our findings may provide new potential therapeutic targets for asthma treatment.

## 1 Introduction

Asthma is a common chronic respiratory disease, affecting more than 300 million people worldwide, and its morbidity and mortality are continuously increasing (1). The pathogenesis of asthma includes chronic airway inflammation and hyperreactivity, bronchoconstriction, mucus overproduction, and airway remodeling (2). At present, mitochondrial dysfunction plays a central role in the pathogenesis of airway remodeling in asthma (3). However, the exact pathogenesis of asthma has not been fully understood.

NLRP3 (NOD-like receptor family pyrin domain containing 3), as an immune sensor of cellular stress, is closely related to the development of asthma. It is reported that the up-regulation of mitochondrial reactive oxygen species (mtROS)-NLRP3 inflammasome signaling pathway in airway epithelial cells promotes airway inflammation, and this effect can be reversed by the antioxidant MitoQ (4). Inhalation of house dust mite (HDM), the common allergen causing asthma (5), can promote NLRP3 inflammasome activation in the lung and specifically induce maturation of caspase-1 and IL-1β in alveolar macrophages (6). HDM can also induce inflammatory cytokine release, pyroptosis, and airway epithelial cell barrier damage in human airway epithelial cells (BEAS-2B) (7). Conversely, NLRP3 inflammasome inhibition could attenuate apoptosis in a contrast-induced acute kidney injury model (8). However, the exact mechanism of NLRP3 inflammasome activation and apoptosis in asthma remains unknown.

Autophagy is strongly associated with airway remodeling in asthma. Elevated levels of autophagy have been detected in sputum granulocytes, neutrophils, and peripheral blood eosinophils of asthmatic patients (9, 10). Double-membrane autophagosomes have been detected in fibroblasts and epithelial cells of asthmatic patients (11). There are higher Beclin 1 and ATG5 expressions and lower p62 expression in asthmatic patients (12). Clearance of damaged mitochondria by mitophagy plays a crucial role in cellular antioxidant defense by maintaining mitochondrial quality and preventing pathological mtROS generation (13), however, in asthma, excessive mitophagy activation is detrimental (14, 15). Mitophagy is mediated by the PTEN-induced kinase 1 (PINK1)-Parkin signaling pathway (16). Amira et al. (17) reported that the levels of *PINK1* and *Parkin* mRNA, inflammatory cytokines, and reactive oxygen species (ROS) were significantly increased in asthmatic patients, with the highest levels in patients with severe atopic asthma. Dimasuay et al. (18) found that Parkin-deficient primary human tracheobronchial epithelial cells and Parkin-knockout mice had decreased release of airway mitochondrial genome (mtDNA) and attenuated level of inflammation under IL-13 or HDM exposure. Cigarette smoke is reported to induce PINK/Parkin-mediated mitophagy in airway epithelial cells and initiate airway inflammation and epithelial-mesenchymal transition (19). Together, autophagy and mitophagy are important for asthma.

DEK is an oncogene located at chromosome locus 6p22.3 and is considered a co-factor of transcription factors (20). Functionally, DEK is involved in chronic inflammation (21). Mor-Vaknin et al. (22) showed that DEK gene deletion or treatment with DEK-targeted aptamers significantly reduced joint inflammation and significantly attenuated the ability of neutrophils to form neutrophil extracellular traps in a mouse arthritis model. The pro-inflammatory chemokine interleukin-8 (IL-8) induces monocyte-derived macrophages to secrete phosphorylated DEK, which can acts as a chemokine to attract neutrophils, CD8^+^ T lymphocytes, and natural killer cells (23). Our previous studies have demonstrated that DEK protein was highly expressed in asthma and DEK-targeting aptamer DTA-64 or miRNA-181b-5p inhibited airway inflammation and airway remodeling in asthma (24, 25). Recently, Zhang et al. (26) found that DEK promoted autophagy in gastric cancer cells through AMPK/mTOR signaling pathway. However, the regulation of PINK1-Parkin-mediated mitophagy by DEK in asthma remains largely unknown.

Herein, we investigated the regulation of DEK on the PINK1-Parkin pathway, NLRP3 inflammasome, and apoptosis in the HDM-induced asthma model. The potential mechanisms were analyzed and discussed. Our findings may provide potential therapeutic targets for patients with asthma.

## 2 Materials and methods

### 2.1 Animals

Female DEK-wild type and DEK-knockout (C57BL/6J-Dek^em1cyagen^) C57BL/6J mice (8-10 weeks old and weighing 20-22 g) were from Yanbian University Health Science Center (Animal License [JI] 2020-00093, Yanji, China) and Saiye (Suzhou) Biotechnology Co., LTD (China), respectively. Mice had free access to food and water and were maintained at room temperature of 22 ± 2°C, relative humidity of 50% to 60%, and light/night cycle of 12h/12h. All animal experiment procedures were performed following the Regulations on the Administration of Laboratory Animals and approved by the Ethics Committee of Yanbian University School of Medicine (SYXK (JI) 2020-0009).

### 2.2 Asthma model establishment, animal treatment, and grouping

**Figure 1:**
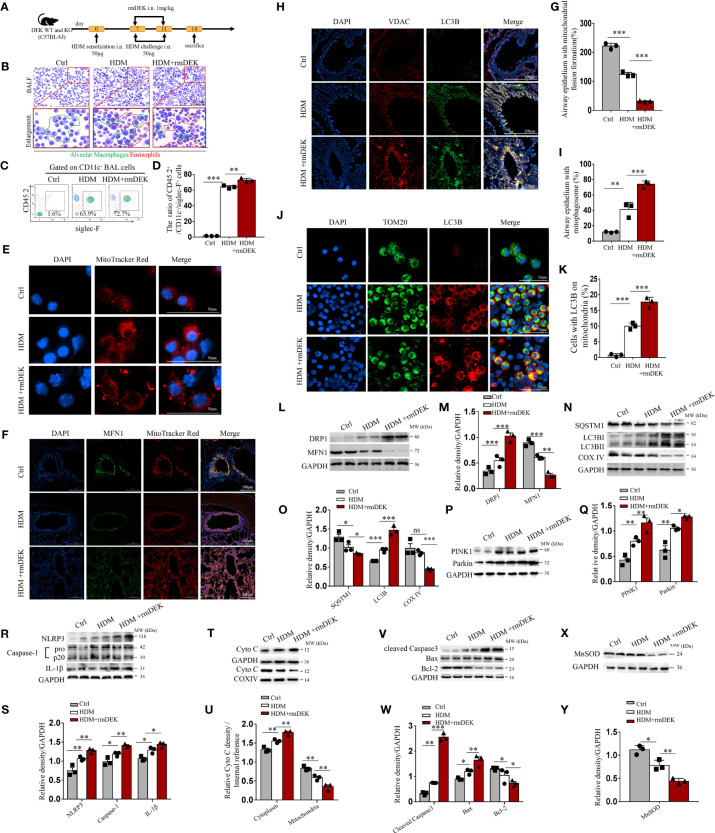
DEK protein induced mitophagy, NLRP3 inflammasome, and apoptosis. **(A)** Schematic diagram of asthma model establishment and rmDEK administration. **(B)** Diff-Quick staining of BAL cells. The red circle indicates eosinophils and the green circle indicates macrophages. Scale bar = 200 μm. **(C, D)** Flow cytometry analysis of CD45.2^+^CD11c^-^siglec-F^+^ cells (eosinophils) and their percentages in BAL cells. **(E)** MitoTracker Red staining of BAL cells to observe mitochondrial morphology. Scale bar = 50 μm. **(F, G)** Representative images and quantification of immunofluorescence of MFN1 and MitoTracker Red. **(H, I)** LC3B and VDAC in airway epithelial cells. Scale bar: 200 μm. **(J, K)** LC3B and TOM20 in BAL cells. Scale bar = 50 μm. Western blot analysis and quantification of **(L–Q)** COX IV, SQSTM1, LC3B I/II, MFN1 , DRP1,PINK1 and Parkin. **(R, S)** caspase-1, IL-1β, and NLRP3; **(T, U)** cytoplasmic and mitochondrial Cytochrome c; **(V, W)** Cleaved caspase-3, Bax, and, Bcl-2; and, **(X, Y)** MnSOD in mouse lung tissues. Data were presented as mean ± SEM. n=3. ns; no statistical difference, *p<0.05, **p<0.01, ***p<0.001.

DEK-wild type (n=18) and DEK-knockout C57BL/6J mice (n=18) were randomly divided into the normal control group, HDM group, and HDM+recombinant DEK (rmDEK) group, respectively. HDM-induced allergic asthma model was established in the HDM group and HDM+rmDEK group as previously described (27, 28). Briefly, on Day 0, sensitization was performed by intranasal (i.n.) instillation of 50 μl HDM (1 μg/μl; #XPB46D3A4, Greer Laboratories, Lenoir, NC, USA) following anesthesia with isoflurane inhalation (**Figure 1A**). On days 7-11, mice in HDM and HDM+rmDEK groups were challenged daily with 50 μg HDM i.n. The mice in the HDM+rmDEK group received intranasal mouse-rmDEK protein (i.n.; 1 mg/kg, CSB-BP766504MO, CUSABIO, Wuhan, China) at 1 h before the HDM challenge to provoke acute exacerbation of allergic airway disease. Mice in the normal control group received an equal volume of PBS.

### 2.3 Sample collection

On day 14, mice were euthanized using 100 mg/kg pentobarbital sodium. The bronchoalveolar lavage fluid (BALF) and the right lung tissues were collected. The BALF was further centrifuged to separate the supernatants and cells.

### 2.4 Cell culture and treatment

Human bronchial epithelial (BEAS-2B) cells were purchased from the Cell Resource Center of Shanghai Institutes for Biological Sciences, Chinese Academy of Sciences (Shanghai, China). Cells were cultured in DMEM (Gibco, Carlsbad, CA, USA) supplemented with 10% fetal bovine serum (Gibco), 100 g/mL streptomycin, and 100 U/mL penicillin at 37°C with 5%CO_2_. For treatment, cells (1×10^5^) were treated with 3-Methyladenine (3-MA, 5 mM), MitoTEMPO (10 μM, HY-112879, MCE, USA), MCC950 (10 μM), or Ac-DEVD-CHO (20 μM) for 4 h before stimulation. For stimulation, human-rmDEK (CSB-BP006710HU, 1 mg/kg, CUSABIO, Wuhan, China) was administered at 1 μg/mL for 24 h, followed by incubation with 200 μg/mL endotoxin-treated HDM (29) (#XPB70D3A2.5/XPB70D3A25, Greer Laboratories) for 24 h. For small interfering RNA (siRNA) transfection, siRNAs specific for human DEK, AAA domain-containing protein 3A (ATAD3A), and dynamin-related protein 1 (DRP1) genes (50 nM; RiboBio, Guangdong, China) were transiently transfected into BEAS-2B cells for 24 h using transfection reagent riboFECT CP (#c10510-05, RiboBio), before stimulation.

### 2.5 Hematoxylin and eosin staining

Lung tissues were fixed, embedded in 10% paraffin, cut into 4 μm sections, and stained with hematoxylin and eosin (#G1120, Solarbio, Beijing, China). The inflammatory cell infiltration in the airways was observed. The pathological damage was scored as follows: 0: normal alveolar structure without inflammatory cell infiltration; 1 point: alveolar structure was normal, with mild damage (<25%) and minimal infiltration of inflammatory cells; 2 points: there was alveolar collapse, with moderate damage (25%~50%) and minimal infiltration of inflammatory cells; 3 points: alveolar structure was not observed with severe damage (>50%) and extensive infiltration of inflammatory cells (30).

### 2.6 Diff-Quick staining

BALF was centrifuged at 150 g for 10 min. The cell pellets were collected and subjected to Diff-Quick staining (#G1541, Solarbio, Beijing, China). Eosinophil morphology was observed under a Slide Scanning Image System (SQS-40R, Shenzhen Shengqiang Technology Co., China).

### 2.7 Immunohistochemical staining

Lung tissue sections were deparaffinized in xylene and dehydrated in graded ethanol. Then, antigen retrieval with citrate was performed. After that, the sections were incubated with primary antibodies against DEK (#29812S, CST, USA), ATAD3A (#A8230, ABclonal, WuHan, China), and DRP1 (#184247, Abcam, USA) overnight at 4°C, followed by incubation with goat anti-rabbit IgG HampL (HRP) (#6721, 1:2000, Abcam, USA). Images were collected with a digital scanning microscopic imaging system and analyzed using Image J software (National Institutes of Health, Bethesda, MD, USA).

### 2.8 ELISA

Manganese superoxide dismutase (MnSOD) expression in lung tissues and BEAS-2B cells was detected with corresponding ELISA kits (#S0103, Beyotime, Shanghai, China).

### 2.9 TUNEL

Apoptosis was measured using the DeadEnd Fluorometric TUNEL System kit (#C1089, Beyotime, Beijing, China), and visualized using Cytation5 (BioTek Instruments, Inc., Winooski, VT, USA).

### 2.10 ROS detection

For detection of total ROS in lung tissues, the lung tissue sections were incubated with ROS-specific fluorescent probe Dihydroethidium (DHE, 10 μM, #S0063, Beyotime) for 5 min at 37°C. For detection of total ROS in cells, BEAS-2B cells were incubated with the DCFH-DA (10 μm/L, #S0033S, Beyotime) for 30 min at 37°C. For mtROS detection, BEAS-2B cells were incubated and stained with MitoSOX (5 μM, #M36008, Thermo Fisher Scientific., Waltham, MA, USA) for 10 min at 37°C. Finally, the fluorescence was observed by Cytation 5 (BioTek) and analyzed using Image J software (National Institutes of Health, Bethesda, MD, USA).

### 2.11 Transmission electron microscopy

BEAS-2B cells were fixed with 2.5% glutaraldehyde for 2 h and washed three times with PBS for 10 min each. The subsequent sample preparation was performed by the Department of Pathology, Yanbian University according to routine procedures. Finally, images were collected under transmission electron microscopy (#HT7700, Hitachi, Japan) at 6000×magnification.

### 2.12 Flow cytometry

To determine the proportion of eosinophils in BALF, cells were stained with PE-Siglec-F antibody (#552126; BD Biosciences, USA), APC-CD45.2 antibody (#558702; BD), and Percp-Cy5.5 CD11c (#45-0114 − 82, Invitrogen, Carlsbad, CA, USA) antibodies for 30 min at 4°C. CD11c^-^CD45.2 ^+^siglecF ^+^ cells were defined as eosinophils. For apoptosis analysis, cell staining was performed using the Annexin-V Apoptosis Detection Kit I (#559763, BD Biosciences, CA, USA). Annexin-V^+^/7-AAD^-^ cells were defined as apoptotic cells. All samples were assessed by Cytoflex flow cytometer (Beckman Coulter, Inc., CA, USA) and analyzed using Cytoexpert 2.4 software.

### 2.13 Immunofluorescence analysis

Immunofluorescence was performed in lung tissue sections, BAL cells, and BEAS-2B cells after fixation and blocking. To label mitochondria, sections and cells were pre-incubated with MitoTracker Red (100 μM, #M7512, Thermo) or MitoTracker Green (100 μM, #M7514, Thermo) probes at 37°C for 30 min, and then incubated with corresponding antibodies. The primary antibodies were as follows: mouse anti-voltage-dependent anion channels (VDAC) antibody (#14734, Abcam); rabbit anti-LC3B (microtubule-associated protein1 light chain 3 beta) antibody (#63817, Abcam); mouse anti-LC3B antibody (#83506S, Abcam); mouse anti-Parkin antibody (#77924, Abcam); rabbit anti-Parkin antibody (# 237469, ABclonal); rabbit anti-mitofusin1 (MFN1) antibody (#57602, Abcam); rabbit anti-translocase of outer mitochondrial membrane 20 (TOM20) antibody (# ab186735, Abcam); rabbit anti-caspase-1 antibody (#ab1872, Abcam); rabbit anti-IL-1β antibody (#AF5103, Affinity); rabbit anti-DRP1 antibody (#8570S, CST); and, anti-ATAD3A antibody (#A8230, ABclonal). For mtDNA measurement, cells were incubated with anti-DNA mouse monoclonal antibody (clone AC-30-10, #61014, 0.5 μg/ml, PROGEN Biotechnik GmbH, R-Biopharm AG, Germany) and rabbit anti-TOM20 antibody (#ab186735, Abcam). The incubation with the primary antibodies was conducted overnight at 4°C. The incubation with secondary antibodies of AlexaFluor488 goat anti-rabbit (#R37118, Life Technologies, USA) and goat anti-mouse IgG HampL Cy3^®^ (#97035, Abcam, USA) was performed for 2 h at room temperature. Finally, the tissue sections and cells were stained with DAPI (# P0131, Beyotime). All images were acquired by Cytation5 and analyzed using Image J software (National Institutes of Health, Bethesda, MD, USA).

### 2.14 Co-immunoprecipitation

Co-immunoprecipitation was conducted with Pierce™ Immunocoprecipitation kit (# 26149, Life). Briefly, BEAS-2B cells were treated with rmDEK (1 μg/ml) for 24 h, collected, and incubated with ice-cold IP Lysis Buffer on ice for 5 min. The lysate was centrifuged at 13,000g for 10 min and the supernatant was collected. The lung tissues (0.5 g) were ground in liquid nitrogen and centrifuged at 13,000g for 10 min. The supernatant was collected and used as input. The crosslinked 4% protein A/G agarose beads were incubated overnight at 4°C with 10 μg of anti-DRP1 antibody (#8570S, Abcam) and anti-IgG antibody (#2729, CST). Then, the supernatants from cells or lung tissues were added and incubated overnight at 4°C for immunoprecipitation. After washing, the samples were subjected to Western blot analysis.

### 2.15 Western blotting

Proteins from cells and lung tissues and the immunoprecipitated proteins were used. After protein concentration determination by NanoPhotometer NP80^®^ Implen (Bavaria, Munich, Germany), 20 μg of total protein was separated using a 12% SDS-PAGE gel. The primary antibodies included anti-DEK (#29812S, CST), anti-MFN1 (#57602, Abcam), anti-DRP1 (#8570S, CST), anti-sequestosome 1 (SQSTM1)/p62 (#211324, Abcam), anti-LC3B (#63817, Abcam), anti-Cytochrome c oxidase IV (COX IV, #16056, Abcam), anti-Cytochrome c (#133504, Abcam), anti-NLRP3 (#15101, CST), anti-caspase-1 (#AB1871, Sigma, St. Louis, MO, USA), anti-IL-1β (#AF5103, Affinity, USA), anti-Cleaved caspase-3 (#9664, CST), anti-Bax (#182858, Abcam), anti-Bcl-2 (#17509, Abcam), anti-PINK1 (#DF7742, Affinity, USA), anti-Parkin (#A0968, ABclonal, USA), anti-MnSOD (#AF5144, Affinity, USA), ATAD3A (#A8230, ABclonal), and anti-GAPDH (#5174, CST). The secondary antibodies were the HRP-goat anti-rabbit antibody (#5151, CST) and the HRP-anti-mouse antibody (#5257, CST). The internal controls were COX IV and GAPDH. The gray values of protein bands were calculated by Quantity One software (BioRad, Hercules, CA, USA).

### 2.16 Chromatin immunoprecipitation-sequencing assay

ChIP assay was performed as previously described (31). Briefly, control (Input) and DEK lentivirus-overexpressing BEAS-2B cells (HA labeled) were crosslinked for 10 min with 1% formaldehyde. After that, the cells were diluted with glycine (125 mM) and lysed with SDS buffer. Then, the obtained chromatin was sonicated and fragmented to 300bp to 500bp. The fragmented DNA (80 μg) was incubated using HA antibody (#ab9110, Abcam) at 4°C overnight and then with protein A/G beads for ChIP. The ChIP-enriched DNA fragments were subjected to DNA sequencing using Illumina (32). The average of peak values of all active regions in the gene were used to calculate the enriched genes. The density of Reads was counted in regions of 5000 bp upstream and downstream of all peak centers, and displayed by histogram and heat map.

### 2.17 Gene ontology enrichment analysis

GO enrichment analysis was conducted by the cluster Profiler R package on the GO database (http://geneontology.org). GO terms with corrected P-values less than 0.05 were considered significantly enriched.

### 2.18 ChIP-quantitative real time-PCR

Total RNA was isolated using RNA Easy Fast Animal Tissue/Cell Total RNA Extraction Kit (#DP451, TIANGEN, Beijing, China) and 1 μg of total RNA was reverse transcribed into cDNA using Fastone-step reverse transcription Kit (#KR118, TIANGEN). Quantitative real-time PCR was performed using SuperReal PreMix Plus (SYBR Green) (#FP205, TIANGEN) and analyzed using Azure cielo 6 system (Azure, Dublin, CA, USA). The primer sequences are listed in **Table 1**. Relative gene expression was calculated using the 2-^ΔΔ^CT method and normalized to GAPDH.

**Table 1:** Primer sequences.

has-ATAD3A-forward	5′-GAAGCGAGCCACCGAGAAGATAAG-3′
has-ATAD3A-reverse	5′-CTCCGAGCACTTCCTCCCGTAG-3′
has-DRP1-forward	5′-ATGCCAGCAAGTCCACAGAA-3′
has-DRP1-reverse	5′-TGTTCTCGGGCAGACAGTTT-3′
has-GAPDH-forward	5′-TGCACCACCAACTGCTTAGC-3′
has-GAPDH-reverse	5′-GGCATGGACTGTGGTCATGAG-3′
mmu-ATAD3A-forward	5′-CAAGCCGCCACATCCTTCACTC-3′
mmu-ATAD3A-reverse	5′-TGCCTTTAGCCTGGTCCCTGTAG-3′
mmu-DRP1-forward	5′-CAGCTGCACTGGCTTCATGACTC-3′
mmu-DRP1-reverse	5′-GTCAACTTGCCATAAACCAGAG-3′
mmu-GAPDH-forward	5′-TCATGGATGACCTTGGCCAG-3′
mmu-GAPDH-reverse	5′-GTCTTCACTACCATGGAGAAGG-3′

### 2.19 Statistical analyses

SPSS 19.0 software (IBM Co., Armonk, NY, USA) was used for data analysis. Data are presented as mean ± standard error of the mean (SEM) of three independent experiments. The significance of differences between the two groups was determined by Student’s t-test. Multiple comparisons were performed using a two-way analysis of variance or Wilcoxon rank sum test. P values<0.05 were considered statistically significant.

## 3 Results

### 3.1 DEK protein activates mitophagy, NLRP3 inflammasome, and apoptosis in mice with HDM-induced asthma

A HDM-induced asthma model was established (**Figure 1A**). In DEK-wild type mice, BALF eosinophils were significantly increased in the rmDEK+HDM group compared with the HDM group (**Figures 1B–D**), and the fragmentation changes of mitochondrial morphology in BAL cells were more evident (**Figure 1E**). Immunofluorescence of lung tissues showed a significant decrease in fusion protein MFN1 (**Figures 1F, G**). The mitophagosomes, which were represented by co-localization of outer mitochondrial membrane protein VDAC and the autophagosome membrane marker LC3B in airway epithelial of lung sections and the co-localization of LC3B and mitochondrial membrane receptor TOM20 in BAL cells, were increased in the rmDEK+HDM group than HDM group (**Figures 1H–K**), indicating that mitophagy is activated by rmDEK. Western blot analysis revealed that fission protein DRP1 increased, MFN1 decreased, autophagosome-related proteins SQSTM1/p62 decreased, LC3B increased, and mitochondrial inner membrane protein COX IV decreased more significantly in the HDM+rmDEK group than that in the HDM group (**Figures 1L–O**). The levels of mitophagy proteins PINK1 and Parkin were significantly increased in the HDM+rmDEK group, further confirming the increased mitophagy (**Figures 1P, Q**). Thus, these results suggest that the rmDEK could down-regulate mitochondrial fusion, and up-regulate mitochondrial fission and mitophagy.

Moreover, the protein levels of NLRP3, Cleaved caspase-1, and mature IL-1β were significantly upregulated in lung tissues of the HDM+rmDEK group (**Figures 1R, S**). The total mitochondrial proteins were extracted and COX IV served as an internal reference for mitochondrial proteins. There was more Cytochrome c in the cytoplasm of the HDM+rmDEK group but significantly less Cytochrome c in mitochondria, suggesting that DEK may aggravate mitochondrial damage (**Figures 1T, U**). The HDM+rmDEK group also had significantly higher levels of pro-apoptotic proteins Cleaved caspase-3 and Bax, but significantly lower levels of the anti-apoptotic protein Bcl-2 (**Figures 1V, W**) and MnSOD (**Figures 1X, Y**). These data imply that DEK may promote NLRP3 inflammasome activation and airway epithelial cell apoptosis in HDM-induced asthmatic mice.

### 3.2 DEK^−/−^ downregulates PINK1-Parkin-mediated mitophagy, NLRP3 inflammasome activation, and apoptosis in mice with HDM-induced asthma

**Figure 2:**
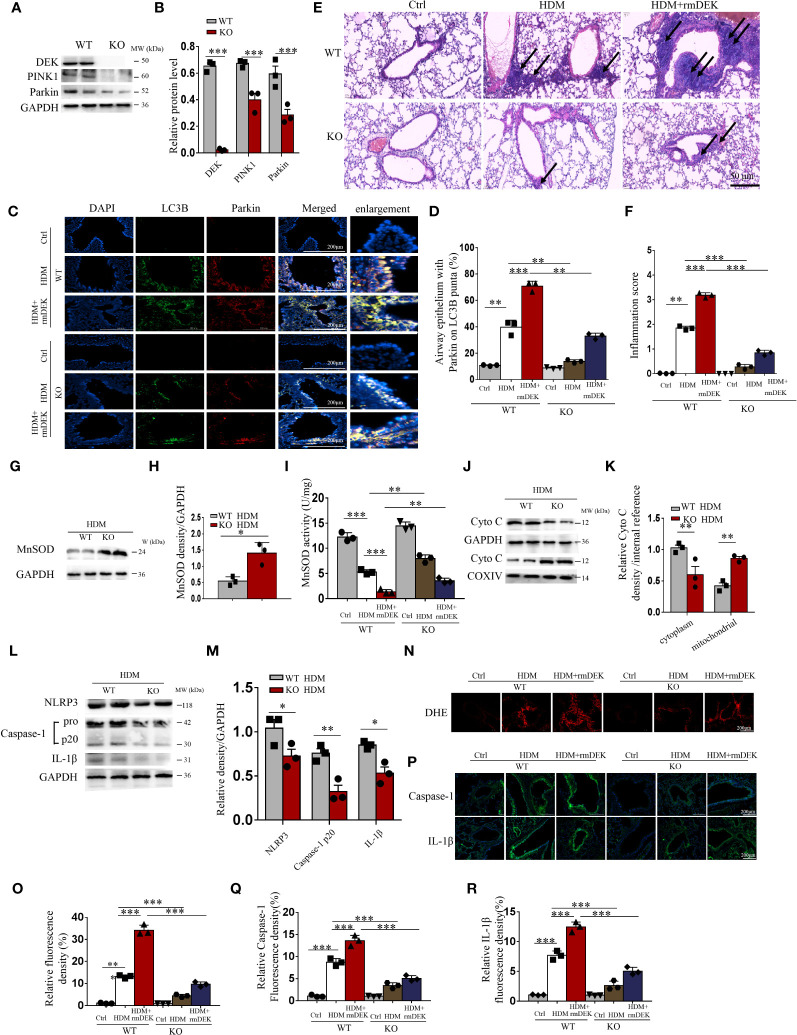
DEK^−/−^ downregulated PINK1-Parkin-mediated mitophagy. **(A, B)** Western blot analysis and quantification of PINK1, DEK, and Parkin in lung tissue from DEK wild type (WT) and DEK knockout mice. **(C, D)** Representative images and quantification of immunofluorescence labeling of LC3B and Parkin. Scale bar = 200 μm. **(E)** HE staining of lung tissues. The arrows indicate the infiltration of inflammatory cells. Scale bar = 50 μm. **(F)** Inflammatory score **(G–I)** Quantification of MnSOD in lung tissue by Western blot and ELISA. Western blot analysis of cytoplasmic and mitochondrial Cytochrome c **(J, K)** as well as NLRP3, caspase-1, and IL-1β of mouse lungs **(L, M)**. **(N, O)** Fluorescence measurement of ROS around the airway using DCFH-DA. Scale bar = 200 μm. **(P–R)** Immunofluorescence staining images and quantification of airway caspase-1 and IL-1β. Scale bar = 200 μm. Data were presented as mean ± SEM. n=3. *p<0.05, **p<0.01, ***p<0.001.

The role of DEK on mitophagy, NLRP3 inflammasome, and apoptosis of asthma model was further verified in DEK-knockout mice. Western blot results showed that DEK, PINK1, and Parkin proteins significantly decreased in mouse lung tissues after DEK knockout (**Figures 2A, B**). Parkin-mediated mitophagy was assessed by immunofluorescence staining of Parkin and LC3B. In DEK-wild type mice, co-localization of Parkin and LC3B was significantly increased by rmDEK treatment compared with HDM alone, and this effect was partially reversed in DEK knock-out mice (**Figures 2C, D**). HE staining showed that inflammatory cell infiltration around the airways was significantly reduced in the HDM + rmDEK group of DEK^−/−^ mice compared with the HDM + rmDEK group of DEK wild-type mice (**Figures 2E, F**). These data reveal that DEK^−/−^ may protect against HDM-induced airway inflammation in asthmatic mice by downregulating PINK1-Parkin-mediated mitophagy.

After intranasal instillation of HDM, the activity of MnSOD decreased in DEK-wild type mice while increased in DEK knockout mice (**Figures 2G–I**), compared with control mice, reflecting that HDM significantly down-regulated MnSOD in the lung tissue of asthmatic mice, and DEK deficiency partially reversed this effect. Moreover, the levels of Cytochrome c were decreased in the cytoplasm but increased in the mitochondria of the DEK-knockout group (**Figures 2J, K**). Similarly, the levels of NLRP3, caspase-1, and IL-1β (**Figures 2L, M, P–R**) were decreased, illustrating the alleviated mitochondrial damage and NLRP3 inflammasome activation. Additionally, DHE staining showed that rmDEK treatment in DEK-wild mice enhanced ROS generation in the airway, while this effect was attenuated in DEK^−/−^ mice (**Figures 2N, O**). Furthermore, Western blot and TUNEL assay found that the HDM+rmDEK group in DEK^−/−^ mice had lower apoptosis levels than in DEK-wild-type mice (**Supplementary Figures S1A–D**). Thus, DEK may regulate airway inflammation in asthma by managing the mtROS, NLRP3 inflammasome, and apoptosis of airway epithelial cells.

### 3.3 Activation effects of DEK on mitophagy, NLRP3 inflammasome, and apoptosis in BEAS-2B cells

**Figure 3:**
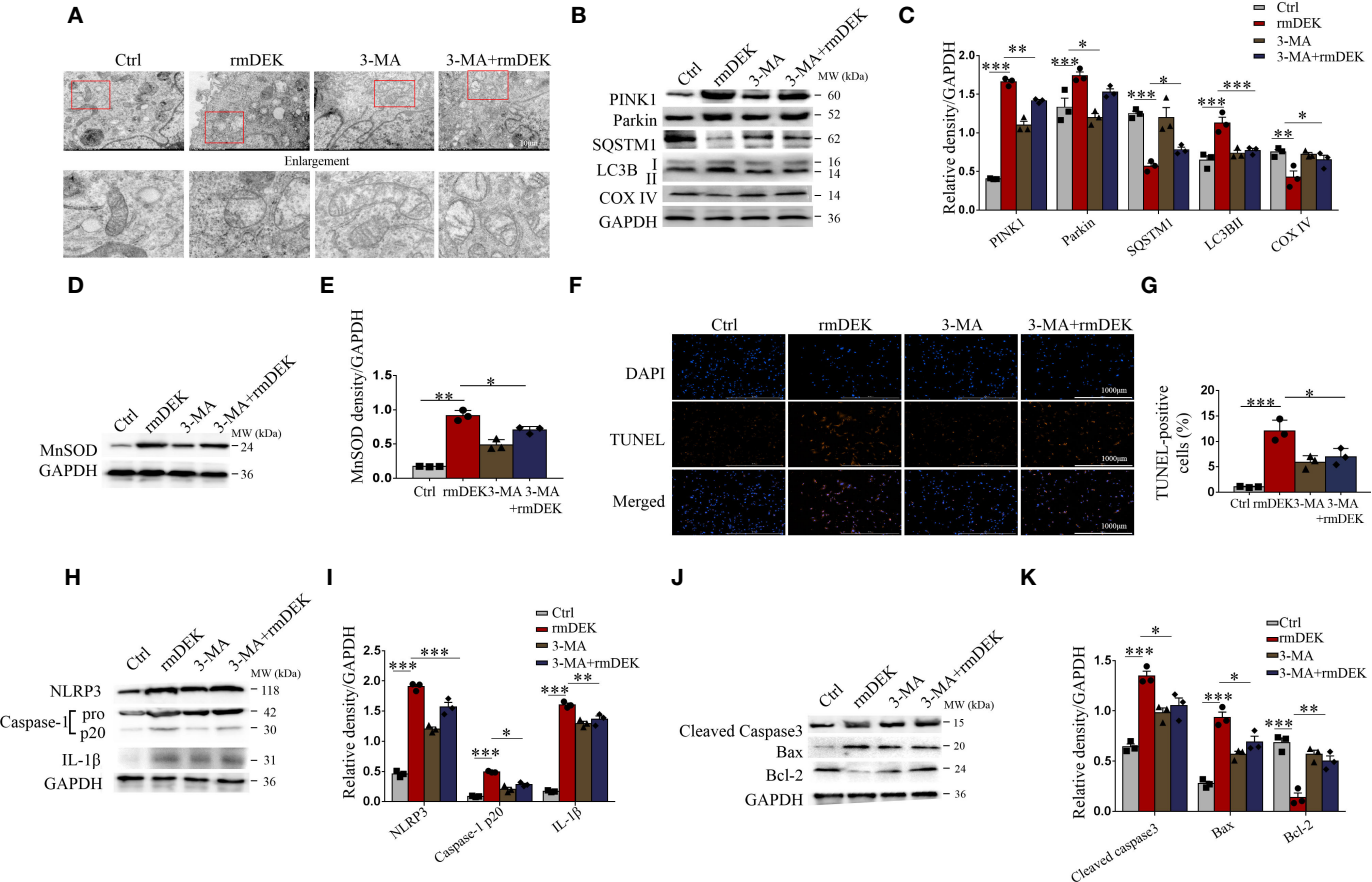
Activation effects of DEK on mitophagy, NLRP3 inflammasome, and apoptosis in BEAS-2B cells. BEAS-2B cells were pretreated with 3-MA (5 mM) for 4 h and then incubated with rmDEK (1 μg/ml) for 24 h. **(A)** Mitochondrial morphology was observed by transmission electron microscopy. Scale bar = 2 μm. **(B–E)** Western blot analysis and quantification of COX IV, SQSTM1, LC3B I/II, PINK1, Parkin, and MnSOD. **(F, G)** Representative images and quantification of TUNEL staining in BEAS-2B cells. Scale bar = 1000 μm. **(H–K)** Western blot analysis and quantification of caspase-1, NLRP3, IL-1β, Cleaved caspase-3, Bax, and Bcl-2. Data were presented as mean ± SEM. n=3. *p<0.05, **p<0.01, ***p<0.001.

The effects of DEK were further analyzed in BEAS-2B cells. Under transmission electron microscopy, mitochondrial swelling and mitochondrial cristae loss were observed in the rmDEK group, which was effectively prevented by 3-MA (**Figure 3A**). Meanwhile, mitochondrial damage was evident followed by exposure to rmDEK. In the rmDEK group, the PINK1, Parkin, and LC3B protein levels were significantly increased, while SQSTM1/p62 and COX IV levels were decreased (**Figures 3B, C**). However, the changes in these proteins were partially reversed by pretreatment with 3-MA. There was higher MnSOD in the rmDEK group but lower MnSOD after 3-MA treatment (**Figures 3D, E**). Compared with the rmDEK group without 3-MA, TUNEL-positive BEAS-2B cells were significantly reduced in the rmDEK group with 3-MA (**Figures 3F, G**). Moreover, the activation of NLRP3 inflammasome and apoptosis was attenuated after 3-MA treatment (**Figures 3H–K**). Thus, in BEAS-2B cells, rmDEK also has partial promotive effects on PINK1-Parkin-mediated mitophagy, NLRP3 inflammasome activation, and apoptosis, and these effects were partially rescued by 3-MA.

### 3.4 Silencing DEK decreases HDM-induced mitophagy in BEAS-2B cells

**Figure 4:**
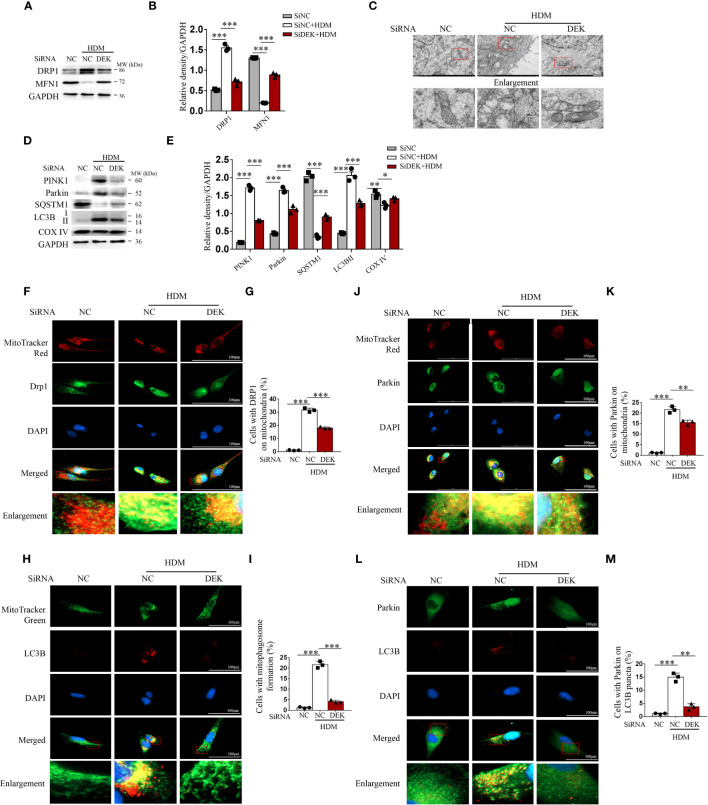
Silencing DEK decreased HDM-induced mitophagy in BEAS-2B cells. The siDEK and negative control siNC were transfected into BEAS-2B cells for 24 h and then treated with HDM (200 μg/mL) for 24 h. **(A, B)** Western blot analysis and quantification of DRP1 and MFN1. **(C)** Mitochondrial morphology was observed by transmission electron microscopy. Scale bar = 2 μm. **(D, E)** Western blot analysis and quantification of PINK1, Parkin, SQSTM1, LC3B I/II, and COX IV. Representative images and quantification of immunofluorescence labeling of DRP1 and MitoTracker Red **(F, G)**; LC3B I/II and MitoTracker Green **(H, I)**; Parkin and MitoTracker Red **(J, K)**; and, LC3BI/II and Parkin **(L, M)**. Data were presented as mean ± SEM. n=3. *p<0.05, **p<0.01, ***p<0.001.

To identify the role and function of DEK in HDM-induced mitophagy in BEAS-2B cells, DEK was silenced using siRNA. Western blot analysis showed that DRP1 was significantly decreased, while MFN1 was significantly increased after siDEK (**Figures 4A, B**). Under transmission electron microscopy, the siDEK group showed reduced mitochondrial swelling and mitochondrial cristae loss compared with the HDM group, indicating attenuated mitochondrial damage (**Figure 4C**). Western blot analysis found that PINK1, Parkin, and LC3B were significantly down-regulated, while the SQSTM1/p62 and COX IV were significantly up-regulated in the siDEK group (**Figures 4D, E**). Consistently, co-localization of DRP1, Parkin, and LC3B with MitoTracker was reduced after siDEK (**Figures 4F–K**). Next, the role of DEK in Parkin-mediated mitophagosome formation following HDM exposure was verified using double-labeling of LC3B and Parkin. As shown in **Figures 4L, M**, a large number of LC3B-labeled mitophagosomes co-localized with Parkin in the HDM+siNC group, but rarely in the HDM+siDEK group. Therefore, DEK silencing reduces HDM-induced mitophagy in BEAS-2B cells. Additionally, the immunofluorescence staining was conducted in both the siDEK group and siNC+rmDEK group to identify the co-localization of Drp1, Parkin, and LC3B with mitochondria. The findings highlighted that the co-localization intensity of Drp1, Parkin, and LC3B on mitochondria was notably enhanced following rmDEK administration, as compared to the siDEK group (**Supplementary Figure S2**). These observations suggest that the decrease in co-localization after siDEK treatment is likely mitigated by rmDEK.

### 3.5 Silencing DEK alleviates the mtROS generation, NLRP3 inflammasome activation, and apoptosis in BEAS-2B cells

**Figure 5:**
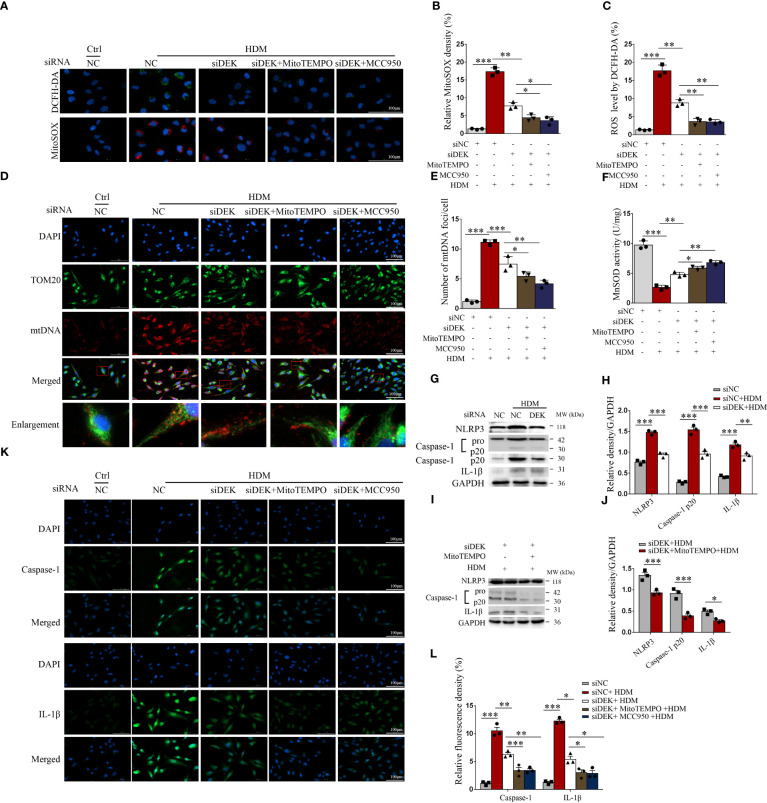
Silencing DEK reduced mitochondrial ROS production and suppressed NLRP3 inflammasome. BEAS-2B cells were transfected with siDEK and negative control siNC for 24 h. Then, the cells were pretreated with MitoTEMPO (10 μM) or MCC950 (10 μM) for 4 h followed by incubating with DMEM containing HDM (200 μg/mL) for 24 h. **(A–C)** Immunofluorescence showed fluorescence intensity of DCFH-DA and mitochondrial ROS (MitoSOX). **(D, E)** Double-labeling of TOM20 and mtDNA. Scale bar = 100 μm. **(F)** MnSOD activity (U/mg) was assessed using ELISA. Western blot analysis and quantification of NLRP3, caspase-1, and IL-1β in cells incubated with HDM following siNC and siDEK **(G, H)**; and NLRP3, caspase-1, and IL-1β in cells stimulating HDM pretreated with or without MitoTEMPO after siDEK **(I, J)**. **(K, L)** Immunofluorescence staining and quantification of caspase-1 and IL-1β in BEAS-2B cells. Scale bar = 100 μm. Data were presented as mean ± SEM. n=3. *p<0.05, **p<0.01, ***p<0.001.

BEAS-2B cells were exposed to HDM before treatment with siDEK, MitoTEMPO (a mitochondria-targeted antioxidant), and, MCC950 (an inhibitor of NLRP3). As shown in **Figures 5A–C**, compared with the control group, the fluorescence intensities of DCFH-DA and MitoSOX were significantly enhanced after exposure to HDM, suggesting that the total cellular ROS and mtROS production are induced by HDM. However, these increases were partially reversed after treatment with siDEK, MitoTEMPO, and MCC950. Then, immunofluorescence co-staining of mtDNA and TOM20 was conducted to examine mtDNA oxidative damage. Damages to the inner and outer membranes of mitochondria may cause the release of large amounts of mtDNA, which may accelerate inflammation and asthma (33). **Figures 5D, E** showed that mtDNA and TOM20 co-localization increased upon HDM stimulation and decreased markedly after siDEK. Furthermore, the activity of MnSOD was increased significantly after siDEK (**Figure 5F**). Western blot revealed that the NLRP3, cleavage of caspase-1, and maturation of IL-1β were activated by HDM, which was further partially rescued by siDEK (**Figures 5G, H**). MitoTEMPO intervention following siDEK further attenuated NLRP3 inflammasome activation (**Figures 5I, J**). Similar effects on caspase-1 and IL-1β were observed by immunofluorescence (**Figures 5K, L**). Additionally, there was an increase in the pro-apoptotic protein cleaved caspase-3 and Bax and a decrease in the anti-apoptotic protein Bcl-2 in BEAS-2B cells after siDEK treatment (**Supplementary Figures S3A, B**). Cleaved caspase-3 inhibition was even more pronounced in cells treated with both MCC950 and siDEK (**Supplementary Figures S2C, D**). Flow cytometry and TUNEL assays demonstrated that Annexin V^+^/7-AAD^-^ early apoptotic cells and TUNEL-positive apoptotic cells were significantly increased after HDM stimulation and significantly decreased after siDEK. The decrease in apoptotic cells was more prominent in cells treated with siDEK and MCC950 or siDEK and caspase-3 inhibitor Ac-DEVD-CHO (**Supplementary Figures S3E–H**). Overall, siDEK could reduce HDM-induced mitochondrial oxidative damage in BEAS-2B cells by reducing mtROS, NLRP3 inflammasome activation, and apoptosis. Additionally, there may be a partial synergistic effect of MCC950 and Ac-DEVD-CHO with siDEK.

### 3.6 DEK regulates DRP1 through ATAD3A

**Figure 6:**
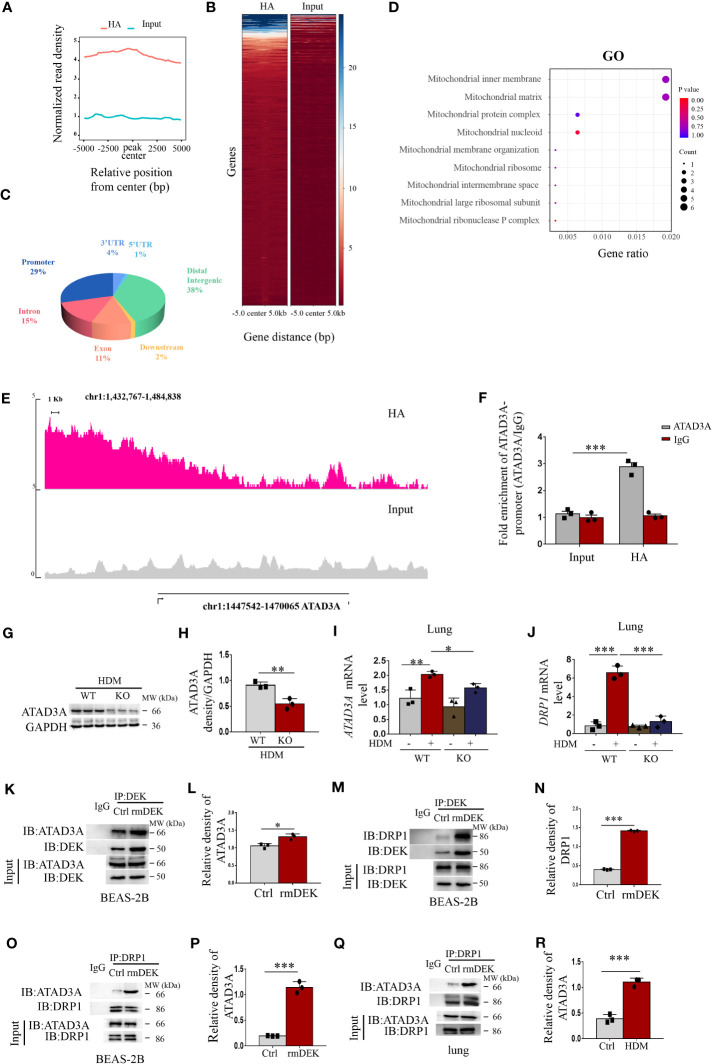
DEK regulated DRP1 via ATAD3A. ChIP-Seq was performed on the control (Input) and DEK overexpressing BEAS-2B cells (HA). **(A)** Peak density histograms following ChIP-Seq. **(B)** Heat map of peak density. **(C)** Statistics of the distribution of the peak region, such as 3′ UTR, 5′ UTR, Distal Intergenic, Downstream, Exon, Intron, Promoter, etc. **(D)** Gene Ontology enrichment analysis after screening using the keyword of mitochondria. **(E)** Peak density of ATAD3A. **(F)** ChIP-quantitative real-time PCR analysis of *ATAD3A* mRNA levels. **(G, H)** Western blot analysis and quantification of ATAD3A in lung tissue of DEK wild type (WT) and DEK knockout (KO) mice pretreated with HDM. **(I)** The expression of *ATAD3A* mRNA level and **(J)** *DRP1* mRNA level in lung tissue. Western blot analysis of ATAD3A, DRP1. and DEK after co-immunoprecipitation in BEAS-2B cells **(K–N)**. Western blot analysis of ATAD3A and DRP1 after co-immunoprecipitation in BEAS-2B cells **(O, P)** and lung tissues **(Q, R)**. Data were presented as mean ± SEM. n=3. *p<0.05, **p<0.01, ***p<0.001.

To further validate DEK modification of mitochondrial dysfunction, we performed ChIP-Seq. The data of control (Input) and DEK lentivirus-overexpressing BEAS-2B cells (HA) were compared. It was found that the peak value, which indicates the enriched genes, in the DEK overexpression group was significantly higher than that of the control group (**Figures 6A, B**). Furthermore, the distribution of the Peak region showed that the enrichment was more obvious in the promoter region, accounting for about 29%, and the 3′ UTR region, accounting for about 4% (**Figure 6C**). GO enrichment analysis of these enriched genes followed by screening using the keyword of mitochondria showed that these genes were significantly enriched in mitochondrial nucleoid, mitochondrial inner membrane signaling, etc (**Figure 6D**), indicating that DEK may significantly affect mitochondrial biology.

ATAD3A, a member of the AAA-ATPase family, is an integral mitochondrial membrane protein that is associated with mtDNA replication and transcription (34). Among the enriched genes, ATAD3A had the most significant difference between control and DEK overexpressing BEAS-2B cells and thus we selected ATAD3A as a DEK-enriched candidate target gene for further investigation. As shown in **Figure 6E**, a clear increase in ATAD3A peak was detected in the DEK-overexpressing group, indicating the enrichment of DEK at the ATAD3A promoter region. This result was further validated by ChIP-real-time quantitative PCR (**Figure 6F**). In mice with HDM-induced asthma, ATAD3A protein and mRNA were reduced in the DEK^-/-^ group (**Figures 6G–I**). These results suggest that ATAD3A may be a downstream target gene of DEK. It has been shown that ATAD3A can interact with DRP1 in Huntington’s disease model, and the disturbance of their interaction inhibits mitochondrial fragmentation and mtDNA damage (34). We found that after DEK knockout, the *DRP1* mRNA level was down-regulated (**Figure 6J**). Co-immunoprecipitation analysis found that the interactions of DEK with ATAD3A or DRP1 in BEAS-2B cells were increased in the rmDEK group in both BEAS-2B cells and lung tissues (**Figures 6K–N**). Furthermore, ATAD3A interacted with DRP1 in BEAS-2B cells and lung tissue, and the interaction was more prominent when cells were exposed to rmDEK or in mice with HDM-induced asthma (**Figures 6O–R**). Taken together, DEK may regulate DRP1 through ATAD3A, thereby regulating mitochondrial function.

### 3.7 Silencing ATAD3A can down-regulate DRP1 induced by HDM

**Figure 7:**
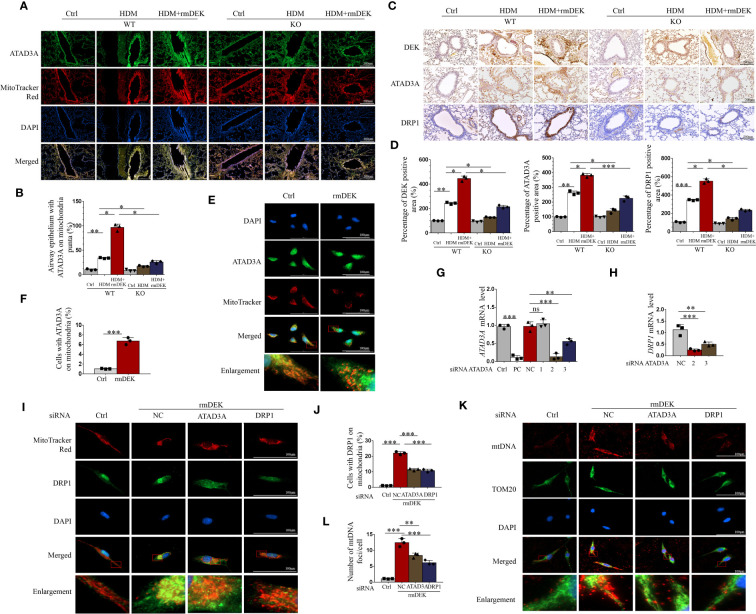
Silencing ATAD3A down-regulated HDM and DEK protein induced DRP1 expression and mitochondrial DNA damage. **(A)** Immunofluorescence showing the co-localization of ATAD3A and MitoTracker Red in lung tissues from DEK wild type (WT) and DEK knockout mice induced by HDM or HDM+rmDEK. **(B)** The intensity of mitochondrial ATAD3A puncta was calculated with Image **(J)** Scale bar = 200 μm. **(C)** Immunohistochemical staining of DEK, ATAD3A, and DRP1 in lung sections of mice. Scale bar = 200 μm. **(D)** The intensity of DEK, ATAD3A, and DRP1 was quantified with Image **(J)**. **(E)** Immunofluorescence showing the co-localization of ATAD3A and MitoTracker Red in BEAS-2B cells pretreated with rmDEK. **(F)** The intensity of mitochondrial ATAD3A puncta was calculated with Image **(J)** Scale bar = 100 μm. **(G)** Expression of *ATAD3A* mRNA levels in BEAS-2B cells transfected with positive control siRNA (siPC), negative control siRNA (siNC), and siATAD3A 1, 2, and 3 for 24 h. **(H)** Expression of *DRP1* mRNA levels in BEAS-2B cells transfected with negative control siNC, siATAD3A, and siATAD3A 3 for 24 h. **(I, J)** Immunofluorescence showing the co-localization of DRP1 and MitoTracker Red in BEAS-2B cells treated with rmDEK (1 μg/mL) for 24 h after transfection with negative controls siNC, siATAD3A and siDRP1. Scale bar = 100 μm; **(K, L)** Immunofluorescence staining of TOM20 and mtDNA. Data were presented as mean ± SEM. n=3. ns=no statistical difference, *p<0.05, **p<0.01, ***p<0.001.

First, immunofluorescence showed that the co-localization of ATAD3A and mitochondria was enhanced after rmDEK intervention in DEK wild-type mice with HDM-induced asthma, and the co-localization was attenuated after in DEK knockout mice (**Figures 7A, B**). Immunohistochemistry of lung tissue showed that DEK, ATAD3A, and DRP1 expression was the highest in the HDM+rmDEK group of DEK wild-type mice and their levels were decreased in DEK knockout mice (**Figures 7C, D**). The above results indicate that ATAD3A and DRP1 are highly expressed in HDM-induced asthmatic mice, and their levels are lowered in mice with DEK knockout.

Cellular immunofluorescence showed that there was co-localization of ATAD3A and mitochondria in BEAS-2B cells after rmDEK treatment (**Figures 7E, F**). To further verify that the regulation of DRP1 by DEK is via targeting ATAD3A, we interfered with ATAD3A using siRNA. The No. 2 siRNA for ATAD3A had the optimal silencing effect on *ATAD3A* mRNA (**Figure 7G**), and it also had the most significant down-regulating effect on *DRP1* mRNA (**Figure 7H**). Immunofluorescence observed that the co-localization of DRP1 and mitochondria was enhanced in BEAS-2B cells after rmDEK treatment and decreased after siATAD3A and siDrp1 (**Figures 7I, J**). Mitochondrial oxidative damage was indicated by co-localization of mtDNA and TOM20 in mitochondria, which was promoted by rmDEK protein and alleviated by siATAD3A and siDrp1 (**Figures 7K, L**). Collectively, DEK may promote mitochondrial damage and dysfunction by regulating ATAD3A and DRP1.

## 4 Discussion

This study is the first to identify a hitherto uncharacterized mechanism, i.e. the DEK/ATAD3A/DRP1 signaling axis, by which DEK promotes PINK1-Parkin mitophagy, NLRP3 inflammasome activation, and aggravates airway inflammation in asthma. Both *in vivo* and *in vitro* experiments revealed that the mitochondrial fusion/fission imbalance, PINK1-Parkin-mediated mitophagy, ROS generation, NLRP3 inflammasome activation, and apoptosis were induced by DEK protein. These effects were partially reversed in DEK knockout mice, by gene silencing, or by the specific inhibitors (MitoTEMPO, MCC950, and Ac-DEVD-CHO). Furthermore, ChIP-seq, ChIP-real-time quantitative PCR, and co-immunoprecipitation showed that DEK could enrich on the promoter region of mitochondrial membrane protein ATAD3A and could regulate its expression, which then could bind to DRP1 and regulate DRP1-mediated mitochondrial dysfunction.

It has been reported that mtROS and mtDNA can trigger the activation of the NLRP3 inflammasome in the airway remodeling of asthma (4). Subsequently, NLRP3 inflammasome could accelerate cell death through the cGAS-STING DNA-sensing pathway (35). Relatively, excessive activation of NLRP3 inflammasome could also induce severe mitochondrial damage, including the release of damaged and improperly repaired mtDNA from mitochondria into the cytoplasm (36). Our results showed that DEK protein activated mtROS production, promoted oxidative mtDNA release, and NLRP3 inflammasome activation *in vivo* and *in vitro*, sufficiently embodied the regulatory role of DEK on the mtROS/mtDNA mediated oxidative stress and NLRP3 inflammasome activation. We have previously shown that IL-8 can induce DEK production in BEAS-2B cells, thereby affecting neutrophil function (24). HDM induces IL-8 production in epithelial cells (37). IL-8 stimulates macrophages to increase endogenous DEK and release DEK into the extracellular space (23). Moreover, DEK secreted by activated synovial macrophages from arthritis patients can be taken up by neighboring cells (38). In addition, exogenous rmDEK is actively endocytosed by small invaginations on the cell membrane via heparan sulfate proteoglycans, then moves into the nucleus, integrates into chromatin, and performs its biological function (38). Therefore, we suggest that endogenous DEK (released into the extracellular space and taken up by neighboring cells) and exogenous DEK (which can enter cells directly) may have similar biological functions, possibly mediated by the IL-8-epithelial-DEK axis. Additionally, NLRP3 inflammasome inhibition could attenuate apoptosis (8). On the contrary, the apoptotic effectors could regulate the NLRP3 inflammasome activation (39). Our results indicate that DEK could regulate apoptosis. DEK protein up-regulated caspase-3 and Bax, while down-regulated Bcl-2 and Cytochrome c, suggesting that DEK protein is closely related to Caspase-mediated apoptosis. Caspase family members are important effectors in apoptosis and can affect chromatin structure and alter intranuclear structural proteins (40). Hua et al. (41) reported that DEK, as a DNA-binding protein, could be induced at the initial stage of apoptosis under low levels of Caspase activation. Lee et al. (42) found that DEK could induce apoptosis in Drosophila through caspase-9- and caspase-3-dependent pathways. In addition to the Caspase family, the relationship of DEK with mitochondrial apoptosis has also been reported. For example, Kim et al. (43) demonstrated that DEK knockdown resulted in decreased Bax levels in HeLa cells, prevented Bax migration from the cytoplasm to the mitochondrial membrane, resulted in Cytochrome C release, and increased the Bcl-2 expression (8).

The mechanism of PINK1-Parkin-mediated NLRP3 inflammasome activation in asthma is largely controversial (44). Zhong et al. (45) indicated that damaged mitochondria in macrophages underwent Parkin ubiquitination, which was specifically recognized by p62, thereby inducing mitophagy and removing NLRP3 inflammasome. In diabetic nephropathy, Chen et al. (46) demonstrated that gene silencing of the autophagy receptor optineurin activated the NLRP3 inflammasome by increasing mtROS in renal tubular epithelial cells after stimulation with high glucose. These findings suggest that mitophagy may have the ability to inhibit NRLP3 inflammasome activation. However, NLRP3 inflammasome activation by excessive mitophagy has also been reported. For example, Jia et al. (47) showed that NaAsO2 upregulated the levels of oxidized mitochondrial DNA and mitophagy, thereby activating NLRP3 inflammasome in the liver of SD rats, and that inhibition of excessive mitophagy using CsA alleviated NaAsO2-induced NLRP3 inflammasome activation and impaired insulin signaling. MiR-423 increased PINK1-mediated mitophagy and NLRP3/Caspase-1/IL-1β inflammasome signaling in HDM and rhinovirus-challenged asthmatic mice (44). Here, we demonstrated that NLRP3 inflammasome activation was inhibited by 3-MA and MitoTEMPO. MitoTEMPO is a widely used mitochondria-targeted antioxidant and can also be used to inhibit mitophagy (48, 49). 3-MA is an autophagy inhibitor that can reduce autophagy-induced apoptosis and inflammation in the OVA-induced airway remodeling model in mice (50). Based on these previous findings and our findings, we conclude that PINK1-Parkin-mediated mitophagy can promote NLRP3 inflammasome pathway activation and apoptosis in HDM-induced asthma. Next, we found that after DEK knockout in mice or DEK silencing in cells, the levels of PINK1-Parkin-mediated mitophagy, NLRP3 inflammasome activation, and apoptosis were down-regulated. It has been shown that DEK is highly expressed in gastric cancer tissues and cell lines, and the knockdown of DEK inhibits autophagy in cells (26). Additionally, the use of the DEK-targeting aptamer DTA-64 has been shown to reduce OVA-induced airway remodeling in asthma through the modulation of various signaling pathways such as Smad2/3, Smad4, p38MAPK, ERK1/2, JNK, and PI3K/AKT/mTOR (24). The above findings are sufficient to suggest that DEK may somehow regulate PINK1-Parkin-mediated mitophagy and NLRP3 inflammasome and that targeting the DEK pathway could be exploited therapeutically in airway inflammation and asthma.

Next, we further used CHIP-seq to explore the key nodes mediating the regulation of DEK on PINK1-Parkin-mediated mitophagy. We found that DEK was enriched on the ATAD3A promoter region, which was further validated by real-time quantitative PCR and Western blot. ATAD3A is a mitochondrial membrane protein that is associated with mitochondrial DNA replication and transcription (51). However, the function of ATAD3A in airway inflammation in asthma is unclear. In our present study, we observed high ATAD3A expression in the lung tissue of HDM-induced asthmatic mice for the first time, which was reduced after DEK knockout. Furthermore, immunofluorescence analysis after rmDEK exposure revealed a slight increase in ATAD3A brightness and significant fragmentation of filamentous mitochondria in BEAS-2B cells and lung tissues. It is concluded that DEK could positively regulate ATAD3A. Furthermore, we found that ATAD3A could interact with DRP1 in DEK protein-induced BEAS-2B cells and HDM-induced asthmatic lungs, and the DRP1-mediated mitochondrial fission was effectively reduced after silencing ATAD3A. Thus, we suppose that DEK may regulate mitochondrial function via modulating ATAD3A. This hypothesis could be supported by other findings. For example, Gilquin et al. (52) suggested that the loss of function of ATAD3A at the contact site between the inner and outer mitochondrial membranes could induce cell fission. The binding of ATAD3A to DRP1 drives mitochondrial division and promotes mitophagy (34). The mechanism may be that ATAD3A interacts with both the inner and outer mitochondrial membranes simultaneously. Therefore, ATAD3A can regulate mitochondrial dynamics at the interface between the inner and outer mitochondrial membrane and is involved in a variety of cellular responses (34). It is well known that the dynamic change of mitochondria mediated by downstream DRP1 is the key factor in activating mitophagy (53). Ren et al. (54) suggested that resveratrol attenuated D-galactose-induced mitochondrial elongation in aging cardiomyocytes by activating Drp1/Parkin/PINK1 signaling. Therefore, the potential role of ATAD3A in mitochondrial dynamics is maintained by interaction with DRP1. The DEK/ATAD3A/DRP1 signaling axis may mediate the effects of DEK on mitophagy and NLRP3 inflammasome in asthmatic airway inflammation.

In summary, this study demonstrates the relationship between DEK and PINK1-Parkin-mediated mitophagy, mtROS, oxidative mtDNA damage, NLRP3 inflammasome, and airway epithelial cell apoptosis. DEK was found to activate NLRP3 inflammasome and apoptosis by promoting PINK1-Parkin-mediated mitophagy, both *in vivo* and *in vitro*. DEK gene knockout, silencing, and targeted inhibitors down-regulated PINK1-Parkin-mediated mitophagy, NLRP3 inflammasome activation, and apoptosis, thereby improving airway inflammation in asthma. The underlying mechanism may be the DEK/ATAD3A/DRP1 signaling axis. Therefore, targeting DEK to downregulate PINK1-Parkin-mediated mitophagy may offer an effective approach for preventing and treating airway inflammation in asthma. However, due to the difficulty in obtaining human lung tissue samples, we were unable to conduct in-depth studies on DEK in human lung tissues. To better reflect clinical reality, future research should consider exploring the expression and function of DEK in human bronchial epithelial tissues, thereby providing new insights and approaches for the diagnosis and treatment of airway inflammatory diseases. In addition, due to technical limitations, we used the global DEK knock-out mice rather than the cell type-specific knock-out mice. This limitation may hinder a comprehensive understanding of DEK’s role in airway inflammation. Therefore, in future studies, we will investigate the effect of DEK in airway epithelial cells using the Cre-LoxP system or other cell-specific knockout methods to reveal its specific role in the pathogenesis of asthma.

## Data availability statement

All data included in this study are available upon request by contact with the corresponding author. The original contributions presented in the study are publicly available. This data can be found here: https://www.ncbi.nlm.nih.gov/geo/query/acc.cgi?acc=GSE243532.

## Ethics statement

The animal study was approved by the Ethics Committee of Yanbian University School of Medicine (SYXK (JI) 2020-0009). The study was conducted in accordance with the local legislation and institutional requirements.

## Author contributions

QB: Conceptualization, Data curation, Investigation, Software, Validation, Writing – original draft. RL: Conceptualization, Methodology, Software, Writing – review & editing. CQ: Data curation, Formal analysis, Writing – review & editing. XH: Investigation, Methodology, Writing – review & editing. DW: Investigation, Methodology, Writing – review & editing. CW: Formal analysis, Software, Writing – review & editing. ZW: Formal analysis, Software, Writing – review & editing. LL: Data curation, Formal analysis, Writing – review & editing. LCL: Data curation, Formal analysis, Writing – review & editing. HP: Data curation, Formal analysis, Writing – review & editing. YS: Conceptualization, Funding acquisition, Software, Writing – review & editing. GY: Conceptualization, Funding acquisition, Project administration, Software, Writing – review & editing.
