# Supplementary material for: DEK deficiency suppresses mitophagy to protect against house dust mite-induced asthma
Source: Front Immunol. 2024 Jan 11;14:1289774. doi: 10.3389/fimmu.2023.1289774 (PMC10808738; doi:10.3389/fimmu.2023.1289774)
Supplement: Supplementary file 1 [file DataSheet_1.docx]

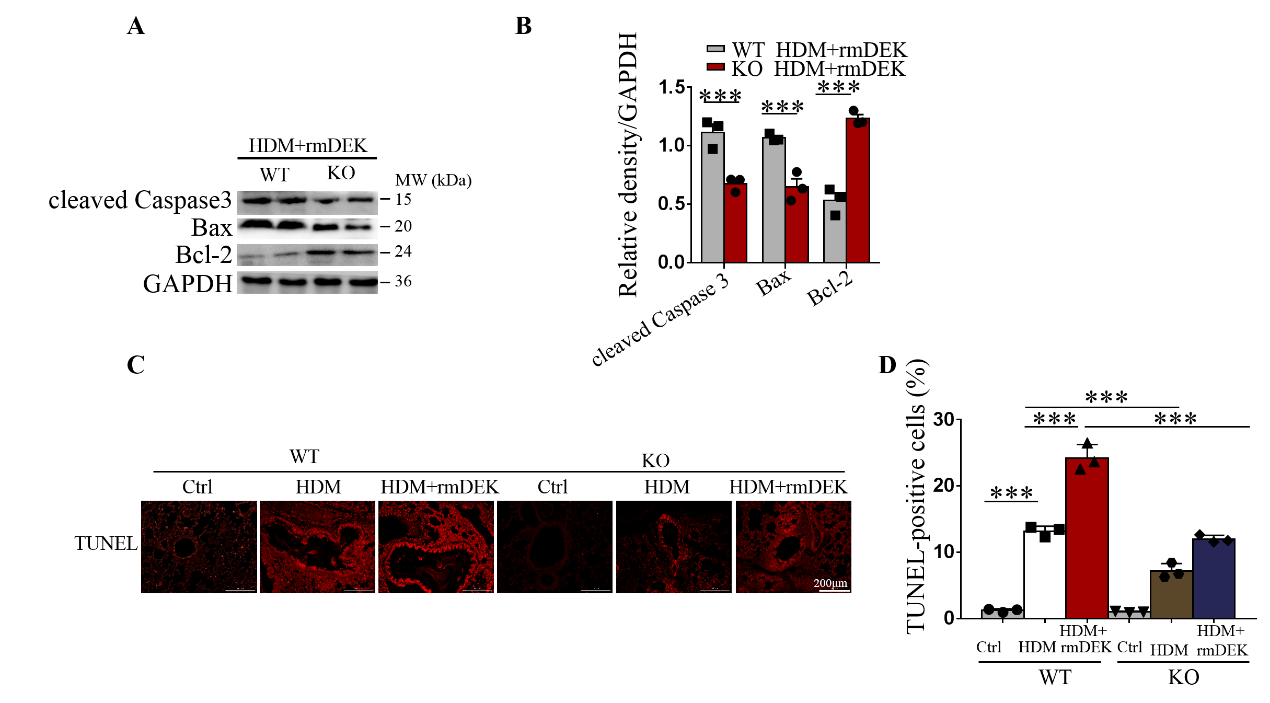


**Figure S1. DEK−/− reduced airway epithelial cell apoptosis in asthmatic mice.**

(A and B) Western blot analysis and quantification of cleaved caspase-3, Bax, and Bcl-2 in lung tissue of DEK wild type (WT) and DEK knockout mice induced by HDM. (C) Apoptosis was assessed by TUNEL staining. (D) Quantification of TUNEL-positive cells in lung tissue sections. Scale bar=200 μm. Data were presented as mean±SEM. n=3. *p<0.05，**p<0.01，***p<0.001.


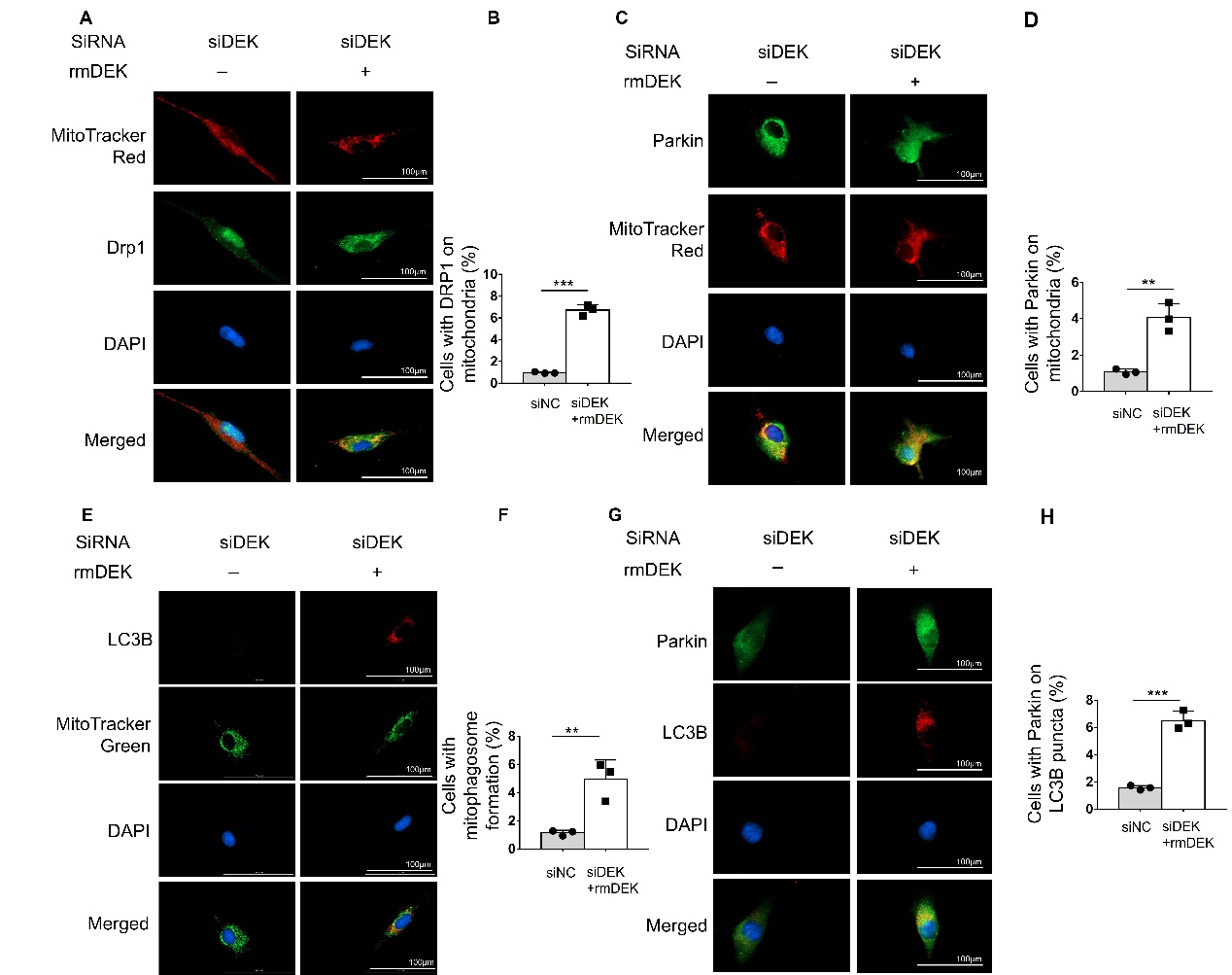


**Figure S2. RmDEK rescued co-localization of Drp1, Parkin and LC3B on mitochondria reduced by siDEK.**

BEAS-2B cells were transfected with siDEK for 24 h and then treated with rmDEK (1 μg/ml) for 24 h. Representative images and quantification of immunofluorescent labeling of DRP1 and MitoTracker Red (A and B); Parkin and MitoTracker Red (C and D); LC3B I/II and MitoTracker Green (E and F); and LC3BI/II and Parkin (G and H). Scale bar = 100 μm Data presented as mean ± SEM. n = 3. p < 0.05, p < 0.01, p < 0.001.


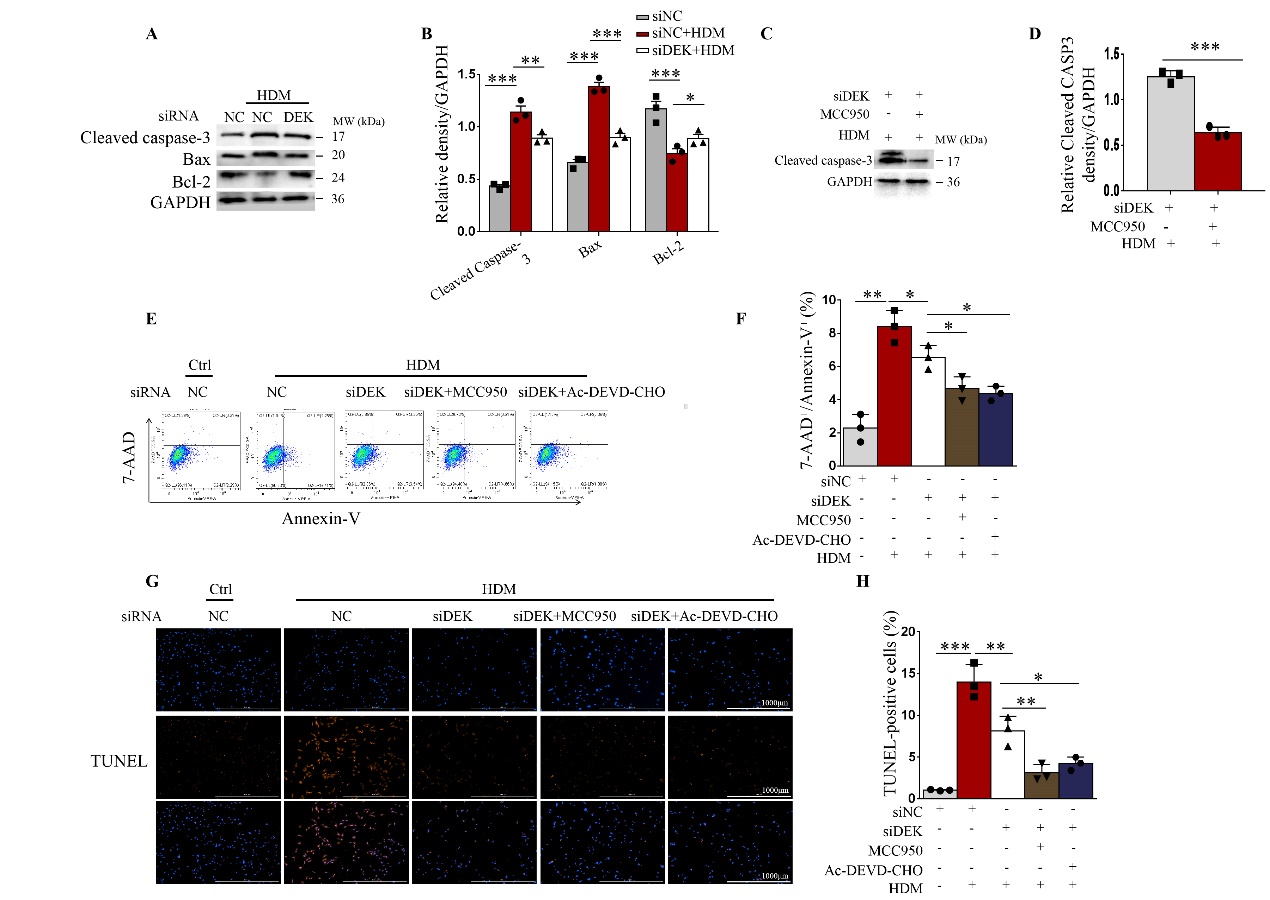


**Figure S3.** **Silencing DEK reduced HDM-induced apoptosis in BEAS-2B cells.**

BEAS-2B cells were transfected with siDEK and negative control siNC for 24 h. Then, the cells were pretreated with MCC950 (10 μM) or Ac-DEVD-CHO (20 μM) for 4 h and incubated in DMEM containing HDM (200 μg/mL) for 24h. (A and B) Western blot analysis and quantification of cleaved caspase-3, Bax, and Bcl-2. (C and D) Western blot analysis and quantification of cleaved caspase-3. (E-H) Representative images and quantification of apoptosis by flow cytometry and TUNEL staining in BEAS-2B cells. Scale bar=1000 μm. Data were presented as mean±SEM. n=3. *p<0.05, **p<0.01, ***p<0.001.
